# Supplementary material for: Novel prognostic determinants of COVID-19-related mortality: A pilot study on severely-ill patients in Russia
Source: PLoS One. 2022 Feb 25;17(2):e0264072. doi: 10.1371/journal.pone.0264072 (PMC8880431; doi:10.1371/journal.pone.0264072)
Supplement: S1 Table — (DOCX) [file pone.0264072.s001.docx]

**S1 Table. The accepted reference values of the routine blood tests parameters.**

| **Parameter** | **Reference values** | **Units** |
| --- | --- | --- |
| RBC count | 3.7-5.4 | 10^12^/l |
| Hemoglobin | 120-140 | g/l |
| WBC count | 3.8-11.8 | 10^3^/μl |
| Neutrophil count | 1.9-8.2 | 10^3^/μl |
| Neutrophils % | 42.7-76.8 | % |
| Lymphocyte count | 1.1-3.8 | 10^3^/μl |
| Lymphocytes % | 16-45.9 | % |
| Monocyte count | 0.2-0.9 | 10^3^/μl |
| Monocytes % | 4.3-11.0 | % |
| Platelet count | 179-408 | 10^3^/μl |
| Total protein | 66-83 | g/l |
| Albumins | 35-52 | g/l |
| Globulins | 20-30 | g/l |
| Total bilirubin | <19 | μM |
| Urea | <7.2 | mM |
| Creatinine | <96 | μM |
| ALT | <34 | U/l |
| AST | <31 | U/l |
| LDH | <247 | U/l |
| CK | <200 | U/l |
| APTT | 25.1-36.5 | S |
| Prothrombin | 78-142 | % |
| INR | 0.64-1.17 | - |
| Fibrinogen | 2.2-4.98 | g/l |
| D-dimer | <0.5 | mg/l |
