# Supplementary material for: Novel prognostic determinants of COVID-19-related mortality: A pilot study on severely-ill patients in Russia
Source: PLoS One. 2022 Feb 25;17(2):e0264072. doi: 10.1371/journal.pone.0264072 (PMC8880431; doi:10.1371/journal.pone.0264072)
Supplement: S2 Table — Reference group is highlighted in bold. MFI–Median Fluorescence Intensity. (DOCX) [file pone.0264072.s002.docx]

**S2 Table. Categorization of biochemical parameters.** Reference group is highlighted in bold. MFI – Median Fluorescence Intensity.

| **Parameter** | **Groups** | | | **Units** |
| --- | --- | --- | --- | --- |
| Dead lymphocytes count | **<100** | >100 |  | 1/μl |
| Dead lymphocytes % | **<1.5** | >1.5 |  | % |
| Early apoptotic lymphocytes | <5% | **5-10%** | >10% | % |
| Late apoptotic lymphocytes | **<0.1%** | >0.1% |  | % |
| CD95+ lymphocytes | <37 | **37-46** | >46 | % |
| CD14+, HLA-Dr+ monocytes | <85% | **>85%** |  | % |
| Akt | **<107** | >107 |  | MFI |
| Bad | **<101** | >101 |  | MFI |
| Bcl-2 | **<20.25** | >20.25 |  | MFI |
| Caspase-8 | **<81.25** | >81.25 |  | MFI |
| Caspase-9 | **<200** | >200 |  | MFI |
| JNK | **<200** | >200 |  | MFI |
| p53 | **<72.25** | >72.25 |  | MFI |
| IL-17 | **<7** | >7 |  | pg/ml |
| PLG | **<20** | >20 |  | μg/ml |
| IL-1α | **<0.3** | >0.3 |  | pg/ml |
| PAI-1 | **<40** | >40 |  | pg/ml |
| TGFβ | **<30** | >30 |  | pg/ml |
| TNFα | **<11** | >11 |  | pg/ml |
| Adiponectin | <5 | **5-10** | >10 | μg/ml |
