# Supplementary material for: Novel prognostic determinants of COVID-19-related mortality: A pilot study on severely-ill patients in Russia
Source: PLoS One. 2022 Feb 25;17(2):e0264072. doi: 10.1371/journal.pone.0264072 (PMC8880431; doi:10.1371/journal.pone.0264072)
Supplement: S3 Table — HR–hazard ratio, CI–confidence interval, Inf–infinite. *–P < 0.05, **–P < 0.01. (DOCX) [file pone.0264072.s003.docx]

**S3 Table.** **Univariable Cox proportional hazards survival analysis models.** HR – hazard ratio, CI – confidence interval, Inf – infinite. * – *P* < 0.05, ** – *P* < 0.01.

| **Parameter** | **HR (95% CI)** | ***P* value** |
| --- | --- | --- |
| **Demographic and clinical characteristics** | | |
| Gender | F: *reference*  M: 1.13 (0.34-3.78) | 0.838 |
| Age, years | 18-39: 0.00 (0.00-Inf)  40-49: 1.18 (0.07-19.52)  50-59: *reference*  60-69: 2.24 (0.23-21.81)  70-79: 0.60 (0.03-10.57)  80+: 2.53 (0.28-22.59) | 0.999  0.909  0.488  0.728  0.406 |
| BMI, kg∙m^-2^ | <30: *reference*  30-34.9: 0.95 (0.28- 3.29)  35-39.9: 0.00 (0.00-Inf)  40+: 0.00 (0.00-Inf) | 0.948  0.999  0.999 |
| Smoking | Non-smoker: *reference*  Smoker: 0.00 (0.00-Inf) | 0.999 |
| CT | 0-2: *reference*  3-4: 4.40 (0.94-20.63) | 0.06 |
| Ventilation | No: *reference*  Yes: 9.86 (2.10-46.23) | 0.004 ** |
| **Hematology results** | | |
| RBC count, 10^12^/l | 0.75 (0.3-1.86) | 0.534 |
| Hemoglobin, g/l | 0.99 (0.95-1.02) | 0.421 |
| WBC count, 10^3^/μl | 1.16 (1.03-1.30) | 0.014 * |
| Neutrophil count, 10^3^/μl | 1.17 (1.03-1.33) | 0.013 * |
| Neutrophil, % | 1.11 (1.01-1.22) | 0.025 * |
| Lymphocyte count, 10^3^/μl | 0.22 (0.04-1.12) | 0.068 |
| Lymphocyte, % | 0.86 (0.75-0.99) | 0.036 * |
| Monocyte count, 10^3^/μl | 0.37 (0.01-19.28) | 0.619 |
| Monocyte, % | 0.74 (0.54-1.01) | 0.056 |
| Platelet count, 10^3^/μl | 1.003 (0.996-1.009) | 0.423 |
| **Clinical biochemical routine blood test results** | | |
| Total serum protein, g/l | 1.01 (0.94-1.09) | 0.807 |
| Albumins, g/l | 0.91 (0.82-1.01) | 0.071 |
| Globulins, g/l | 1.15 (1.02-1.30) | 0.021 * |
| Total bilirubin | 0.99 (0.96-1.02) | 0.534 |
| Urea, mM | 1.09 (1.04-1.15) | 0.001 ** |
| Creatinine, μM | 1.005 (1.002-1.009) | 0.006 ** |
| ALT, U/l | 1.002 (1.0004-1.003) | 0.013 * |
| AST, U/l | 1.001 (1.0002-1.001) | 0.010 * |
| LDH, U/l | 1.001 (1.0002-1.001) | 0.005 ** |
| CK, U/l | 1.003 (1.001-1.005) | 0.002 ** |
| APTT, s | 1.01 (0.998-1.022) | 0.093 |
| Prothrombin, % | 0.99 (0.96-1.01) | 0.391 |
| INR | 1.17 (0.66-2.07) | 0.591 |
| Fibrinogen, g/l | 1.26 (0.95-1.67) | 0.105 |
| D-dimer, mg/l | 1.26 (1.02-1.55) | 0.032 * |
| **Laboratory blood findings** | | |
| Dead lymphocytes, count/μl | 1.009 (1.003-1.013) | 0.001 ** |
| Dead lymphocytes, % | 2.45 (1.21-4.96) | 0.013 * |
| Early lymphocyte apoptosis | 0.91 (0.77-1.06) | 0.216 |
| Late lymphocyte apoptosis | 3.08 (0.01-882.66) | 0.696 |
| CD95+ lymphocytes, % | 0.98 (0.94-1.02) | 0.370 |
| CD14+/HLA-Dr+ monocytes, % | 0.97 (0.95-0.99) | 0.005 ** |
| AKT | 1.00 (1.00-1.00) | 0.593 |
| BAD | 1.01 (1.00-1.01) | 0.063 |
| BCL2 | 0.90 (0.79-1.02) | 0.107 |
| Caspase 8 | 1.03 (0.99-1.07) | 0.128 |
| Caspase 9 | 1.00 (1.00-1.01) | 0.258 |
| JNK | 1.00 (1.00-1.00) | 0.510 |
| P53 | 0.97 (0.94-1.02) | 0.230 |
| IL-17, pg/ml | 1.03 (1.01-1.05) | 0.001 ** |
| IL-1α, pg/ml | 1.07 (1.02-1.13) | 0.005 ** |
| PLG, μg/ml | 1.01 (0.87-1.17) | 0.916 |
| PAI-1, pg/ml | 0.96 (0.91-1.01) | 0.104 |
| TNFα, pg/ml | 1.07 (0.95-1.20) | 0.263 |
| TGFβ, pg/ml | 1.00 (1.00-1.00) | 0.159 |
| ADP, μg/ml | 0.88 (0.73-1.06) | 0.263 |
