# Supplementary figures and images for: Novel prognostic determinants of COVID-19-related mortality: A pilot study on severely-ill patients in Russia
Source: PLoS One. 2022 Feb 25;17(2):e0264072. doi: 10.1371/journal.pone.0264072 (PMC8880431; doi:10.1371/journal.pone.0264072)

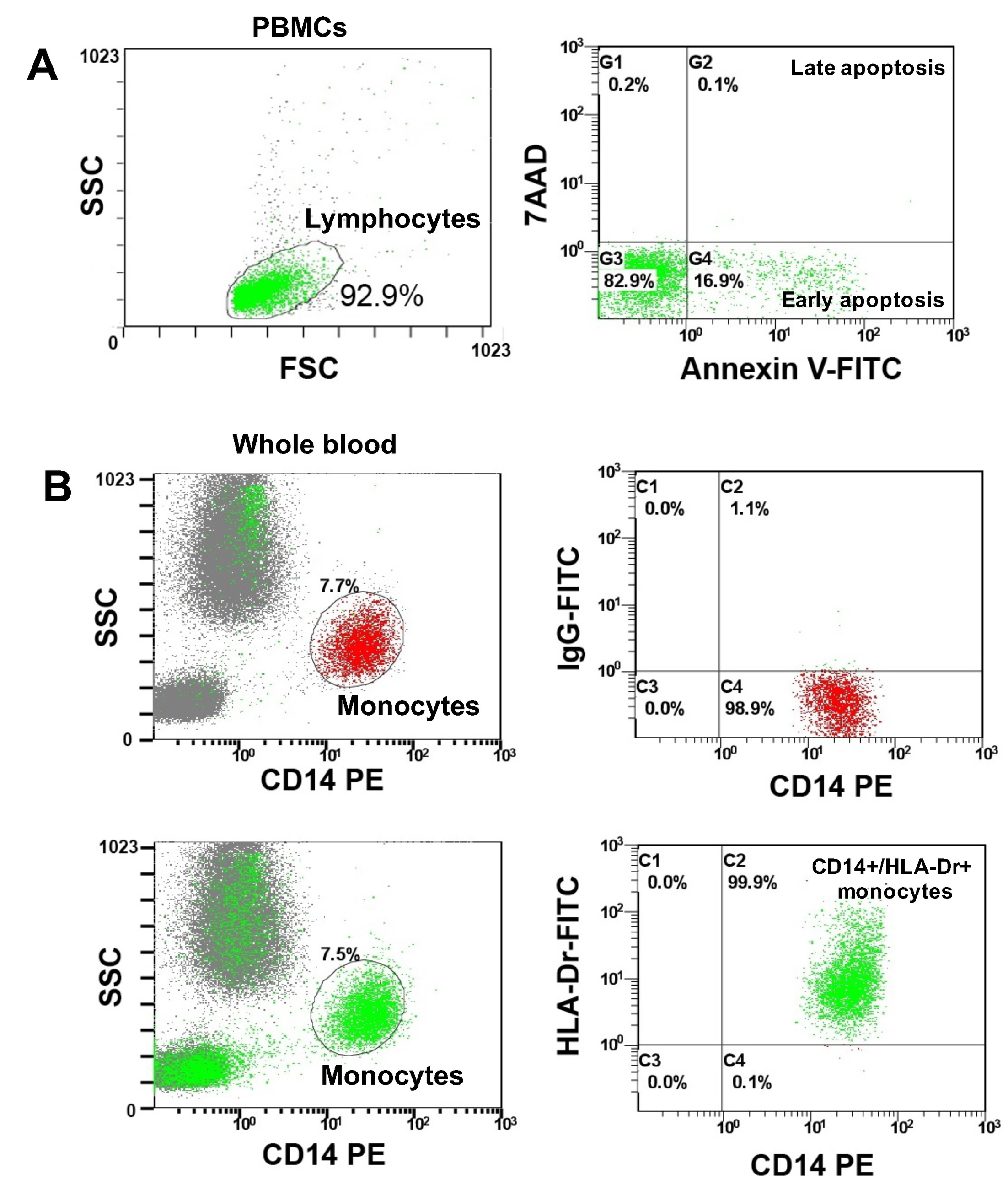

Supplement: S1 Fig — A–Representative flow cytometry graphs showing the gating strategy used to assess lymphocyte apoptosis. Forward scatter (FSC) vs. side scatter (SSC) plot applied to gate the lymphocyte cell population and remove the debris. Annexin-FITC-channel vs. 7AAD-channel plot used to gate early (Annexin V+/7AAD-) and late (Annexin V+/7AAD+) apoptotic cells in lymphocyte population. B–Representative flow cytometry graphs showing the gating strategy used to evaluate the percentage of CD14+/HLA-Dr+ cells in monocyte cell population from the whole blood. Isotypic non-immune IgG (IgG1 Mouse-FITC Isotype Control, A07795, Beckman Coulter) was applied as a control. (TIF) [file pone.0264072.s004.tif]

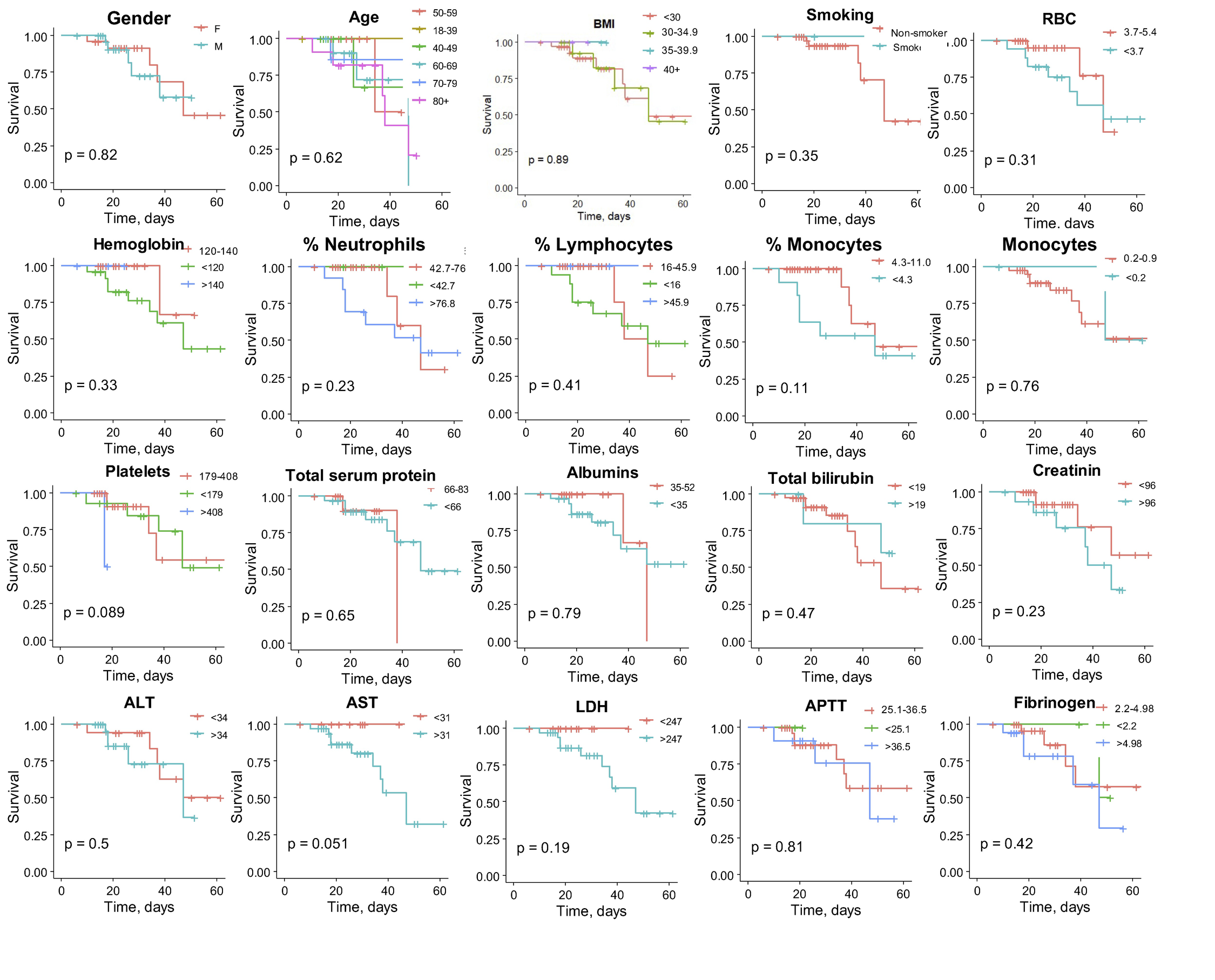

Supplement: S2 Fig — The fraction of survival is expressed on the y-axis, while time (days) of the observation period is expressed on the x-axis. Vertical traits indicate censored data (hospital discharge). P values of log-rank tests are indicated for each graph. Reference group is shown in red. (TIF) [file pone.0264072.s005.tif]

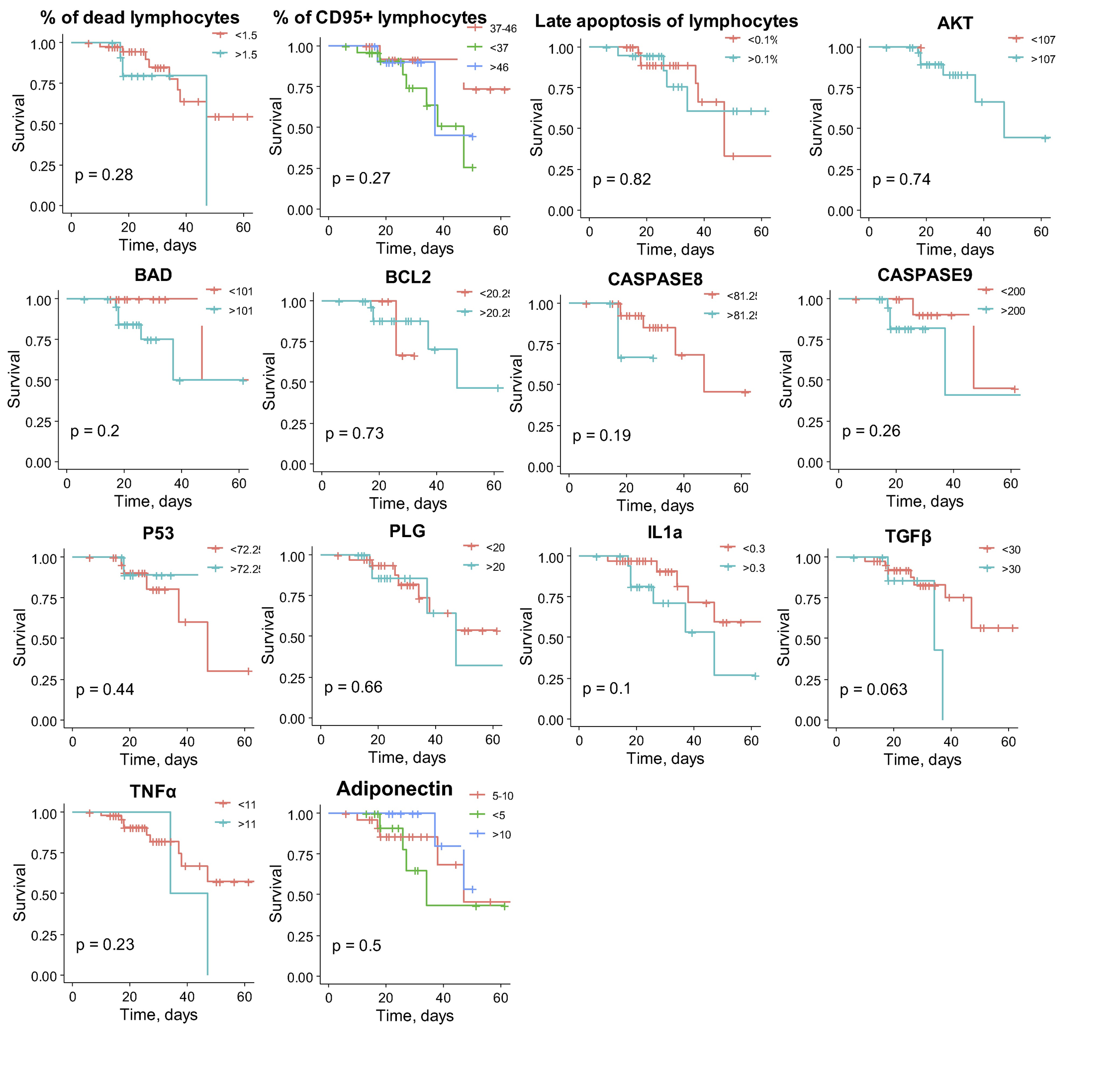

Supplement: S3 Fig — The fraction of survival is expressed on the y-axis, while time (days) of the observation period is expressed on the x-axis. Vertical traits indicate censored data (hospital discharge). P values of log-rank tests are indicated for each graph. Reference group is shown in red. (TIF) [file pone.0264072.s006.tif]
