# Supplementary material for: Novel prognostic determinants of COVID-19-related mortality: A pilot study on severely-ill patients in Russia
Source: PLoS One. 2022 Feb 25;17(2):e0264072. doi: 10.1371/journal.pone.0264072 (PMC8880431; doi:10.1371/journal.pone.0264072)
Supplement: Attachment — Submitted filename: Response to Reviewers R2.docx [file pone.0264072.s008.docx]

|   **LOMONOSOV MOSCOW STATE UNIVERSITY**  **FACULTY OF MEDICINE**  119991 Lomonosovsky av., 27/1  Moscow, Russia  Tel: (495) 932-88-14.  Fax: (495) 939-24-23  E-mail: info@fbm.msu.ru | To: The Editorial Board  ***PLOS ONE***  **7^th^ of January**, 2022 |
| --- | --- |

Dear Dr. Hirst,

We would like to thank the Editorial board for the opportunity to resubmit the revised manuscript to *PLOS ONE* journal. Please, find the cover letter with the answer to the Reviewer’s comment and rebuttal.

The authors would like to thank the Reviewers for their attentive reading and unbiased reviewing of our manuscript, as well as for their comments and discussions.

We have been very concerned about the fate of our manuscript since the revision process took so long with the initial submission being on the 10^th^ of June, while until the 28^th^ of September we received no comments from the Reviewers. Overall, the review process has already taken about 6 months. Reviewer’s comments from the last revision comprised the only recommendation from the Reviewer #1, which has been appropriately considered by us and the corresponding changes has been made.

**Review Comments to the Author:**

Reviewer #1: The discussion section in the revised manuscript seems not have been restructured and rewrote according to the authors' response to my first comment, and the sentence "Gender … several parameters of routine blood tests were not statistically significant" is not removed.

***We restructured the Discussion section to the best of our understanding of the Reviewer’s comments.***

***Concerning the laboratory immunological parameters, we underscored the importance of IL-17 and PAI-1 measurements that were significantly associated with survival by Kaplan-Meier and Cox proportional hazards estimates. We also removed the phrase "Gender … several parameters of routine blood tests were not statistically significant" from the discussion section.***

***Prognostic value of various parameters is canonically evaluated using both, univariable Kaplan-Meier and multivariable Cox regression survival analyses (i.e. PMID: 34675225, PMID: 31901133). Our study has certain limitations as mentioned in the last part of the discussion sections. Due to the total sample size and the event number (deaths) of the study, we considered important to discuss the parameters that are significant by Kaplan-Meier survival analysis and non-significant by Cox proportional hazards survival analysis model. Multivariable analysis (Cox model) was used to reveal independent risk factors, however due to the current experimental settings and the limited statistical power of Cox model this can result in non-significance of the parameters identified by Kaplan-Meier estimates. The aim of the present study was to highlight the potentially relevant prognostic factors that can be further investigated in detail with larger patient cohorts defining practical issues and future perspective.***

***We have been confused by the following Reviewer’s comment concerning “relevant” and “decisive” factors, as we were unable to find these terms in Statistical Analysis literature. However, we revised the conclusions in a more accurate and correct way as recommended by the Reviewer.***

Our scientific team would appreciate if you can promote the review process to run smoothly and fast. The manuscript contains powerful data on early stratification of the patients in ICU and new prognostic markers of severe COVID-19, which can be urgently enrolled into clinics. Due to the spread of new COVID-19 strains worldwide our data could be indispensable for COVID-19 research and treatment.

With best regards,

Ksenia A. Rubina, Doctor of Sciences, PhD,

Head of Laboratory of Morphogenesis and Tissue Reparation, Faculty of Medicine, Lomonosov Moscow State University,

Moscow, Russian Federation

Mobile tel. +7 (903) 728-88-21

Email: kseniiarubina1971@gmail.com
